# Diagnostic value of presepsin for severe diabetic food ulcers: A prospective observational study

**DOI:** 10.1097/MD.0000000000044819

**Published:** 2025-10-03

**Authors:** Osman Baspinar, Derya Kocer, Hasan Bakkal, Oguzhan Sitki Dizdar

**Affiliations:** a Department of Internal Medicine, Kayseri City Training and Research Hospital, Kayseri, Turkey; b Department of Biochemistry, Kayseri City Training and Research Hospital, Kayseri, Turkey; c Department of Internal Medicine and Clinical Nutrition, Kayseri City Training and Research Hospital, Kayseri, Turkey.

**Keywords:** amputation, C-reactive protein, diabetic food ulcers, presepsin, procalcitonin

## Abstract

Diabetic food ulcers (DFU) are a significant complication for patients with type 2 diabetes mellitus. Presepsin is a new biomarker that can offer greater specificity in detecting bacterial infection. So, the study assessed its use in predicting amputation risk in patients with DFU at admission. This prospective study had 2 groups: DFU patients and a control group of type 2 diabetes mellitus patients. DFU patients were staged using the Wagner classification. A presepsin test and review of clinical records were conducted at admission. Seventy-eight patients were included, 49 of whom were male (62.8%). Median age was 70. Sixty-three were in the DFU group, 15 in the control group (no DFU). Increased presepsin levels were observed in patients with DFU, along with elevated C-reactive protein (CRP) and white blood cell (*P* < .001). Patients who underwent amputation procedures had elevated levels of several inflammatory parameters, including procalcitonin, fibrinogen, white blood cell and presepsin, while CRP levels remained unchanged. In regression analysis, the only significant risk factor for severe infection that required amputation was higher Wagner stage. The optimum presepsin cutoff point of ≥ 1020 pg/mL had the highest sensitivity (100%) and specificity (57.1%) for identifying amputation, with an area under the curve of 0.829 (*P* = .003). In contrast, CRP, procalcitonin and fibrinogen had unsatisfactory diagnostic capability for amputation, with area under the curve values of 0.674, 0.600, and 0.714 (*P* = .111, *P* = .359, and *P* = .050, respectively). Presepsin may serve as an effective marker for severe infection in patients with DFU. Integrating presepsin and clinical score interpretations can predict severe infections requiring amputation.

## 1. Introduction

Diabetic food ulcers (DFU) represent a significant complication among patients with type 2 diabetes mellitus (T2DM). They are characterized by a range of conditions affecting the lower extremities, including vascular obstructions, persistent foot infections, ulcers, and deep tissue destruction.^[1,2]^ It is estimated that between 15% and 25% of patients with T2DM will experience DFU.^[3,4]^ DFU and associated amputation represent the predominant causes of morbidity and mortality. Despite the utilization of various techniques, including debridement, infection control, blood glucose regulation, and vascular opening, in the treatment of DFU, these wounds often fail to heal effectively, especially in late cases, ultimately resulting in amputation or mortality.^[5]^ It is therefore of great importance to detect complications at an early stage. If the risk stratification of DFU can be obtained earlier in patients with T2DM, it will be possible to reduce the rates of hospitalization, disability and mortality. However, it is possible that inflammatory biomarkers, which are used for early assessment of DFU, including white blood cell (WBC) count, procalcitonin (PCT), and C-reactive protein (CRP), may be elevated in the context of noninfectious conditions.^[6,7]^ Accordingly, there is a clear need for the exploration of new biomarkers.

Presepsin, a fragment of the lipopolysaccharide (LPS)-binding protein complex receptor (CD14), is released into the circulation when monocytes are activated following binding with LPS and LPS-binding protein. It has been identified as a novel biomarker with the potential to offer greater specificity for bacterial infection.^[8]^ A number of studies have indicated that a cutoff point of > 600 pg/mL is an effective predictor of bacterial infection.^[9]^ Nevertheless, the clinical utility of presepsin remains a topic of debate, as its superior predictive capacity for bacterial infection compared to PCT was observed in relatively small cohorts.^[9,10]^ Additionally, the majority of large-scale studies did not include an adequate number of culture-proven bacterial infections, potentially limiting the generalizability of their findings.^[11–13]^

The relationship between presepsin levels and DFUs is a subject on which there is a paucity of data. To date, the sole investigation undertaken was a retrospective, single-center study utilizing a small sample size and lacking the inclusion of a control group (no DFU).^[14]^ This study found some evidence that presepsin may be an early predictor of severe infections in patients with DFUs that require amputation.^[14]^ Therefore, the objective of this study was to assess the potential of presepsin in predicting the risk of amputation in patients with DFU at the time of hospital admission with a prospective design and to compare T2DM patients with no DFU. Additionally, it was intended to evaluate whether presepsin can provide more accurate prognostic information than conventionally determined parameters, namely, WBC count, CRP, and PCT levels. In order to ascertain the clinical usefulness of presepsin, its performance in predicting culture-proven bacterial infection was evaluated.

## 2. Materials and methods

### 2.1. Patients and design

The prospective study was conducted between June 2022 and October 2023 at a tertiary research hospital. The study comprised 2 groups: a DFU group and a control group, which consisted of patients with T2DM but without DFU. Patients in DFU group were staged in accordance with the Wagner classification system, with DFU defined as a full-thickness skin break of at least Wagner stage I.^[15]^ Patients with other types of diabetes, chronic organ failure (e.g., renal and liver failure), cerebrovascular diseases, malignancy, rheumatologic disease or who were pregnant, as well as those with infectious diseases other than DFU, were excluded from the study. Patients in the control group exhibited no additional diseases other than T2DM. The following criteria were used to diagnose osteomyelitis: a previous medical consultation with a qualified medical practitioner, imaging tests that revealed osteomyelitis, or a postsurgical diagnosis of osteomyelitis. The term “minor amputation” was defined as any amputation distal to the ankle joint, whereas “major amputation” was defined as any amputation through or proximal to the ankle joint.^[16]^

The study was approved by the Institutional Review Board (2019/381) and performed in accordance with the Declaration of Helsinki. Informed consent was obtained from each participant enrolled in the study.

### 2.2. Data collection

A comprehensive review of clinical records was conducted, encompassing the following data points: age, sex, results of wound culture, length of hospital stay, date of amputation if applicable, amputation area, and mortality status. At the time of admission, pertinent preexisting clinical data, such as antidiabetic medications and a history of peripheral artery disease, was meticulously documented. The utilization of vacuum-assisted closure therapy, hyperbaric oxygen or growth factors, which have been demonstrated to play a significant role in regulating the complex process of diabetic wound healing, were documented. A detailed record was also kept of the treatment modalities applied, including antibiotic therapy, debridement, as well as major and minor amputations.

A series of wound cultures were conducted on samples obtained from diabetic foot ulcerations. The definition of “culture-proven bacterial infection” is the isolation of pathogens from potential clinical specimens.

The following routine laboratory measurements were performed: fasting plasma glucose, estimated glomerular filtration rate, creatinine, alanine transaminase, aspartate transaminase, glutamyl transpeptidase, triglyceride, total cholesterol, low-density lipoprotein cholesterol, high-density lipoprotein cholesterol (HDL-C), fibrinogen, uric acid, magnesium, phosphorus, albumin, lactate dehydrogenase, hemoglobin, platelets, and WBC.

### 2.3. Measurement methods

The presepsin test was conducted at the time of admission to the hospital for all patients included in the study. Blood samples were taken from the patients in a seated position and after a 20 minutes rest following 12 hour of fasting. Serum specimens of patients and controls were stored at −80°C until assayed. Serum presepsin levels were determined using sandwich enzyme immunoassay method (range: 31.2–2000 pg/mL; MyBioSource Human Presepsin ELISA Kit, San Diego). Presepsin level was analyzed according to the manufacturer’s instructions and expressed as pg/mL. The concentrations of the samples were calculated through calibration curves obtained from study standards with known levels.

Serum CRP and PCT levels were analyzed by immunoturbidimetric and ECLIA methods on Cobas c702 (Cobas, Roche Diagnostics, Mannheim, Germany) and Cobas e601 (Elecsys, Roche Diagnostics, Mannheim, Germany) respectively.

### 2.4. Statistical analyses

Statistical analyses were performed using SPSS 18.0 software (SPSS Inc., Chicago). Continuous variables were expressed as the mean ± standard deviation or median (interquartile ranges). The normality and the homogeneity of the data were evaluated using Shapiro–Wilk test and the Levene test, respectively. The differences between the 2 groups were analyzed using either the Student *t* test or the Mann–Whitney *U* test, depending on the variables under consideration. Pearson or Spearman correlation were used to evaluate the association of continuous variables according to normality of the data. Binary logistic regression analysis was conducted to detect the independent effect of variables on the presence of severe infection requiring amputation in patients with DFU. The receiver operation characteristic curve (ROC curve) was used to assess presepsin to predict severe infection requiring amputation. *P* < .05 was considered statistically significant.

## 3. Results

A total of 78 patients were included in the study, 49 of whom were male (62.8%). The median age was 70 years (range 42–95 years). A total of 47 patients were using insulin; 18 were using oral antidiabetic medication; and 13 were using a combination of insulin and oral antidiabetic medications. A total of 63 patients were enrolled in the DFU group, while 15 patients constituted the control group, who did not have DFUs. Within the DFU group, 1 patient received hyperbaric oxygen treatment, and 3 patients received growth factors. Meanwhile, a total of 10 patients were administered vacuum-assisted closure therapy. A total of 50 patients were found to have positive wound cultures in the DFU group. The most prevalent microorganisms identified were *Staphylococcus aureus, Proteus mirabilis*, and *Streptococcus agalactiae*.

### 3.1. Comparison between DFU group and control group

The demographic and laboratory data were presented in Table 1. There were no differences between the DFU and control group regarding age and sex. Statistically significant elevations (nearly 2-fold) in presepsin levels were observed in patients within the DFU group, a finding that was concomitant with elevated levels of CRP and WBC (*P* < .001 for both). Albumin and hemoglobin levels were lower in DFU group (*P* = .017 and < .001; respectively). Despite the absence of any statistically significant intergroup variation in glycemic parameters (fasting plasma glucose, HbA1C), a marked elevation in lipid parameters was observed in the control group.

**Table 1:** The comparison of demographic and laboratory data between DFU group and control group.

	DFU group (n = 63)	Control group (n = 15)	*P* value
Age, yr	70 (17)	68 (9)	.970
Sex, M/F, noun (%)	41 (65)/22 (35)	8 (53)/7 (47)	.553
eGFR, mL/dk/1.73 m^2^	81 (40)	92 (20)	.399
Creatinine, mg/dL	0.9 (0.1)	0.8 (0.2)	.216
Presepsin, pg/mL	1320 (504)	660 (550)	<.001
CRP, mg/L	112 (81.9)	4.2 (3.5)	<.001
FPG, mg/dL	174 (97)	149 (102)	.849
HbA1C, %	10.3 (3.8)	9 (2.6)	.088
Total cholesterol, mg/dL	140 (64)	193 (39)	<.001
LDL-C, mg/dL	75 (36)	119 (29)	.002
HDL-C, mg/dL	32 (15)	45 (8)	<.001
Triglyceride, mg/dL	140 (113)	194 (108)	.283
Albumin, g/L	3.2 (1.2)	4.1 (1)	.017
AST, U/L	16 (6)	16 (8)	.325
ALT, U/L	15 (10)	17 (16)	.092
Uric acid, mg/dL	4.7 (2.4)	4.4 (2)	.936
Phosphorous, mg/dL	3.1 (0.9)	3.4 (0.8)	.265
WBC, 10³/µL	13.3 (6)	7.2 (2)	<.001
Hemoglobin, g/dL	11.9 (2.7)	14.4 (2.1)	<.001
Platelet count, 10³/µL	336 (102)	248 (89)	.003

ALT = alanine transaminase, AST = aspartate transaminase, CRP = C-reactive protein, DFU = diabetic food ulcers, eGFR = estimated glomerular filtration rate, F = female, FPG = fasting plasma glucose, HDL-C = high-density lipoprotein cholesterol, LDL-C = low-density lipoprotein cholesterol, M = male, WBC = white blood cell.

### 3.2. Comparison of cases involving severe infections requiring amputation with those that did not in DFU group

Thirty-eight of these patients underwent amputation procedures. There were no significant differences observed between the 2 groups with respect to age or sex, as indicated by the presence or absence of amputation (Table 2). Similarly, the 2 groups exhibited no statistically significant difference with regard to the length of hospital stay, the incidences of osteomyelitis and positive wound culture or the mortality rate. The peripheral artery disease was found to be higher in the amputation group, although no statistically significant differences were observed between the 2 groups. Moreover, a number of inflammatory parameters exhibited elevated levels in the amputation group. These included PCT, fibrinogen, WBC, and presepsin, with CRP levels being unchanged. The Wagner stage was found to be significantly higher in the amputation group. Additionally, HDL-C levels were found to be significantly lower in this group.

**Table 2:** Comparison of the cases that underwent amputation and those that did not in the DFU group.

	Amputation (n = 38)	No amputation (n = 25)	*P* value
Age, yr	71 (15)	64 (24)	.171
Sex, M/F, noun (%)	25 (66)/13 (34)	16 (64)/9 (36)	.884
Peripheral artery disease, noun (%)	15 (41)	4 (16)	.076
Wagner stage	4 (2)	2 (2)	**<.001**
Positive wound culture, noun (%)	33 (87)	17 (68)	.136
Length of hospital stay, day	18.5 (10.5)	17 (18.5)	.482
Osteomyelitis, noun (%)	6 (16)	3 (12)	.674
Mortality, noun (%)	3 (8)	1 (4)	.535
eGFR, mL/dk/1.73 m^2^	79 (41)	92 (43)	.211
Creatinine, mg/dL	0.9 (0.5)	0.8 (0.6)	.598
Procalcitonin, mcg/L	0.14 (0.15)	0.08 (0.15)	**.009**
Fibrinogen, mg/L	7550 (1808)	5730 (2410)	**.044**
Presepsin, pg/mL	1.53 (0.61)	1 (0.39)	**<.001**
CRP, mg/L	115.5 (73)	83.3 (115)	.136
FPG, mg/dL	178 (100)	168 (100)	.227
HbA1C, %	10.6 (2.8)	9.5 (4.3)	.763
Total cholesterol, mg/dL	127 (46)	158.6 (82)	.701
LDL-C, mg/dL	68 (34)	80 (62)	.409
HDL-C, mg/dL	31 (16)	38 (17)	**.047**
Triglyceride, mg/dL	155 (114)	121 (76)	.059
Albumin, g/L	3.2 (1.3)	3.2 (0.98)	.308
AST, U/L	16 (14)	16 (8)	.768
ALT, U/L	13 (8.8)	15 (9.5)	.456
Uric acid, mg/dL	4.6 (2.4)	4.8 (2.5)	.734
Phosphorous, mg/dL	3.3 (0.9)	3.1 (0.9)	.218
Magnesium, mg/dL	1.9 (0.3)	1.8 (0.6)	.262
WBC, 10³/µL	13.9 (4.1)	10.1 (8.2)	**.028**
Hemoglobin, g/dL	12 (2.6)	11.3 (3.3)	.916
Platelet count, 10³/µL	353 (131)	328 (115)	.177

Bold indicates the *P*-values that were below .05 threshold. ALT = alanine transaminase, AST = aspartate transaminase, CRP = C-reactive protein, DFU = diabetic food ulcers, eGFR = estimated glomerular filtration rate, F = female, FPG = fasting plasma glucose, HDL-C = High-density lipoprotein cholesterol, LDL-C = low-density lipoprotein cholesterol, M = Male, WBC = white blood cell.

A logistic regression analysis was conducted into the effects of demographic, clinical, and laboratory parameters on the development of severe infection that required amputation (Table 3). It was only the variables that demonstrated a statistically significant association in the simple logistic regression model (i.e., higher Wagner stage and presepsin, lower HDL-C, and the presence of peripheral artery disease) that were included in the multiple logistic regression model. In multiple logistic regression analysis, the only significant risk factor associated with severe infection that required amputation was higher Wagner stage.

**Table 3:** Risk factors associated with severe infection requiring amputation in patients with DFU according to univariate and multiple logistic regression analysis.

Risk factors	OR	95% CI	*P*
Univariate analysis			
Age	1.035	0.991–1.082	.123
Sex	1.082	0.376–3.111	.884
Peripheral artery disease	0.279	0.080–0.980	**.046**
Wagner stage	8.399	2.963–23.812	**<.001**
Positive wound culture	0.322	0.091–1.137	.078
Osteomyelitis	0.727	0.164–3.222	.675
eGFR	0.988	0.967–1.009	.270
Procalcitonin	5.077	0.685–3762.30	.061
Fibrinogen	1	1–1.001	.113
Presepsin	221.5	12.821–3827.6	**<.001**
CRP	1.003	0.996–1.009	.437
HbA1C	1.047	0.848–1.293	.670
HDL–C	0.940	0.891–0.991	**.022**
WBC	1.062	0.964–1.169	.227
Multiple analysis			
Peripheral artery disease	0.476	0.047–4.849	.531
Wagner stage	10.034	3.105–32.427	**<.001**
HDL-C	0.926	0.836–1.025	.136
Presepsin	16.343	0.241–1109.8	.194

Bold indicates the *P*-values that were below .05 threshold. CI = confidence interval, CRP = C-reactive protein, eGFR = estimated glomerular filtration rate, DFU = diabetic food ulcers, HDL-C = High-density lipoprotein cholesterol, WBC = white blood cell.

### 3.3. The role of presepsin for severe infection that required amputation in DFU group

Levels of presepsin were elevated in patients who died (1630 vs 1290 pg/mL), although this elevation did not attain statistical significance (*P* = .544). Similarly, patients with positive wound cultures and osteomyelitis exhibited higher presepsin levels (1320 vs 1210 pg/mL and 1320 vs 1300 pg/mL, respectively), though these differences were not statistically significant (*P* = .709 and *P* = .969, respectively). Patients with Gram-positive bacterial infections exhibited similar presepsin values to those with Gram-negative bacterial infections (1270 and 1340 pg/mL, respectively; *P* = .946). Patients who underwent major amputation (n = 16) exhibited a significantly higher presepsin level in comparison to those who underwent minor amputation (1730 vs 1320 pg/mL; *P* < .001).

**Figure 1.**
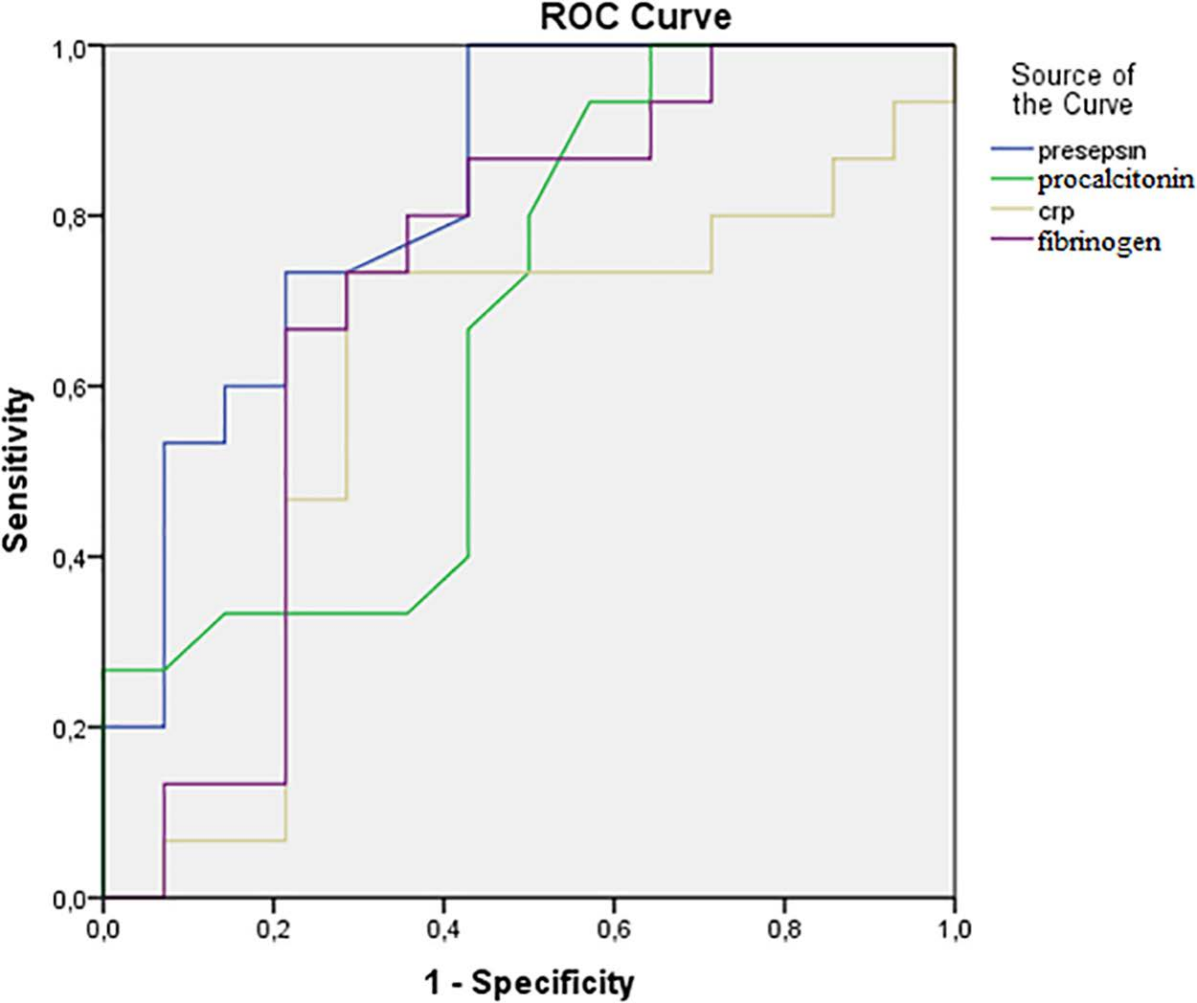
ROC curve for the diagnostic ability of serum the presepsin, CRP, procalcitonin, and fibrinogen levels in identify amputation at admission. CRP = C-reactive protein, ROC = receiver operation characteristic.

The capacity of the serum biomarkers presepsin, CRP, PCT, and fibrinogen to identify amputation was evaluated through the utilization of ROC curves and the calculation of the area under the curve (AUC). ROC curve analysis revealed that the optimum presepsin cutoff point of ≥ 1020 pg/mL had the highest sensitivity (100%) and specificity (57.1%) for the identification of amputation (Fig. 1) with an AUC of 0.829 (95% confidence interval: 0.677–0.980; *P* = .003). In contrast, CRP, PCT, and fibrinogen exhibited unsatisfactory diagnostic capability for amputation, with AUC values of 0.674, 0.600, and 0.714, respectively (*P* = .111, *P* = .359, and *P* = .050, respectively).

In order to ascertain the correlation between elevated presepsin levels and more severe DFU, in addition to related variables, in patients admitted to hospital, correlation analysis were performed. Correlation analysis revealed a significant relationship between the presepsin level and the Wagner score (*R* = 0.684, *P* < .001). However, this relationship did not extend to the length of hospital stay or the operation day following hospitalization, for which no significant correlation was observed (*r* = −0.007, *P* = .955; *r* = −0.201, *P* = .158, respectively). Furthermore, a lack of statistically significant correlation was identified between presepsin and creatinine, as well as estimated glomerular filtration rate (*r* = 0.105, *P* = .361; *r* = −0.177 *P* = .121).

## 4. Discussion

The expeditious identification of bacterial infections and the subsequent determination of their worse prognosis are of paramount importance to the initiation of antimicrobial therapy and the amelioration of clinical outcomes in DFUs. Despite the fact that a considerable number of studies have demonstrated that presepsin is a reliable inflammatory marker for infectious diseases,^[11,17–19]^ only a single study with a limited number of patients has examined the role of presepsin in predicting amputation in patients with DFUs.^[14]^ In the present study, in patients with DM with DFU, presepsin values were found to be significantly higher than those observed in T2DM patients without DFU, and this elevated levels in DFU group were particularly pronounced in those who had undergone amputation. Nevertheless, the findings of the study revealed that, at the time of hospital admission, no inflammatory parameters, including presepsin, were capable of effective prediction of amputation in patients with DFU in a logistic regression analysis. The Wagner stage did possess predictive value for amputation. The existence of a correlation between presepsin and Wagner score serves to emphasize the significance and usefulness of presepsin in the context of DFU.

The presence of DFU biomarkers, which are indicative of the underlying pathophysiology of diabetic wounds, has the potential to facilitate a more comprehensive understanding of DFU. This enhanced knowledge can contribute to the development of improved clinical diagnostic procedures, disease prevention strategies, disease progression prediction models, and treatment evaluation tools for DFU. The WBC level, CRP level, PCT, and fibrinogen levels have been identified as biomarkers for diabetic foot infections.^[20–22]^ These biomarkers are investigated as a means of predicting the severity of DFU. CRP levels are elevated in patients with DFU; however, it has been established that PCT is a more effective diagnostic tool than CRP alone.^[23,24]^ The level of PCT is significantly elevated in infected DFU, and in comparison to CRP, PCT demonstrates higher sensitivity and specificity for the detection of infection in DFU.^[25]^ In our study, the PCT demonstrated a marked increase in the amputation group; however, this increase was not observed in the CRP. Nevertheless, given the findings of studies demonstrating the capacity for PCT elevation in noninfectious conditions, efforts have been underway to identify an alternative ideal biomarker.^[6,7]^ In a study of 207 patients, Endo et al examined the clinical usefulness of presepsin in discriminating between bacterial and nonbacterial infections (including systemic inflammatory response syndrome) by comparing it with PCT and IL-6.^[26]^ The results of this study demonstrated that presepsin exhibited superior sensitivity and specificity in detecting bacterial infections when compared with PCT and IL-6. Furthermore, the measurement of presepsin concentrations has been demonstrated to be a valuable tool in the assessment of the severity of infection and the monitoring of clinical responses to therapeutic interventions.^[27]^ In the study conducted by Ha et al on DFU, the levels of WBC, CRP, PCT, and presepsin were found to be significantly elevated in the amputation group.^[14]^ The present study’s findings are consistent with these results and PCT, fibrinogen, WBC, and presepsin levels were all found to be significantly elevated in the group with severe infections requiring amputation.

In the present ROC curve analysis, the AUC value of presepsin for predicting amputation has been found to exceed those of both PCT and CRP, with a value of 0.829. Conversely, the AUC values for CRP and PCT in predicting amputation were found to be lower (0.674 and 0.600, respectively). Additionally, the diagnostic capability of these biomarkers for amputation did not reach statistical significance. This finding aligns with the results of previous studies that have demonstrated the superiority of the AUC value of presepsin in predicting bacteremia over that of PCT.^[28]^ Kondo et al meta-analysis of 3.012 patients from 19 studies yielded evidence suggesting comparable diagnostic accuracy of PCT and presepsin in detecting bacterial infection (AUC: 0.84 and 0.87, respectively).^[29]^ Nevertheless, the studies incorporated within this analysis exhibited considerable heterogeneity. With regard to the subject with DFU, this superiority for presepsin represents a novel finding in the extant literature. It is important to note that this superiority was not endorsed by preceding research on DFU.^[14]^

The optimum presepsin cutoff point of ≥ 1020 pg/mL had the highest sensitivity (100%) and specificity (57.1%) for the identification of amputation. In the study conducted by Park et al, the optimal cutoff value of presepsin for diagnosing bacteremia was determined to be 1028.5 pg/mL, a finding that aligns with the results of the present study.^[30]^ This values are significantly higher compared to other studies, which reported cutoffs for presepsin that ranged from 675 pg/mL for predicting severe infections necessitating amputation to 729 pg/mL for diagnosing bacteremia.^[14,18]^ However, small sample size, patients with noninfectious etiologies or who had no bacterial infections may contribute to lower presepsin levels in some studies. It is possible that the retrospective cross-sectional study design, the characteristics of the single-center study, and the patient diversity (which included hospitalized patients) may have had an influence on the cutoff value of presepsin.

Despite the postulation that presepsin levels can serve as a diagnostic tool for distinguishing Gram-negative bacteria, the extant literature offers no definitive answer to the question of whether presepsin can differentiate between Gram-positive and Gram-negative bacterial infections.^[26,31]^ The present study found that the difference in presepsin levels between Gram-positive and Gram-negative bacterial infections was not statistically significant.

Despite the documented precedent of a positive correlation between presepsin and both creatinine and GFR, suggesting the necessity of incorporating renal function into the assessment of presepsin,^[32]^ our own research did not reveal a significant correlation between presepsin and renal parameters.

Inadequate glycemic control has been demonstrated to heighten the probability of diabetic foot complications. Nevertheless, a meta-analysis revealed no substantial correlation between HbA1c and diabetic foot amputation.^[33]^ Similarly, the present study did not demonstrate a correlation between glycemic parameters and severe DFU that necessitated amputation.

Future research endeavors must prioritize the evaluation of presepsin in patients with DFU and undertake prospective investigations to ascertain its association with clinically significant outcomes. The persistence of elevated levels of presepsin may offer clinicians a useful clinical indication to consider continuation of antibiotic therapy for patients with DFU. A subsequent investigation should be conducted to determine whether the implementation of a presepsin-based antibiotic prescription strategy is associated with significant decreases in the duration of antibiotic treatment, the length of hospital stay, and the costs of hospitalization for patients with DFU. A further subject of interest is the impact of antidiabetic agents on presepsin levels, with particular attention given to drugs that demonstrate anti-inflammatory properties.

It is important to note that the present study is subject to a number of limitations. Firstly, it should be noted that the study was conducted in a single center, which may limit the generalizability of the results to other populations. Secondly, serial measurements of presepsin during the period of hospitalization were not performed.

In conclusion, presepsin may serve as a useful marker for severe infection in patients with DFU. Despite the fact that no inflammatory parameters, including presepsin, exhibited superiority in predicting amputation in patients with DFU when compared to the Wagner score, presepsin demonstrated the most effective diagnostic capability for amputation among the evaluated biomarkers. The integration of interpretations of inflammatory indicators and clinical scores will facilitate the prediction of severe infections necessitating amputation.

## Author contributions

**Conceptualization:** Derya Kocer.

**Formal analysis:** Derya Kocer

**Resources:** Osman Baspinar, Hasan Bakkal, Oguzhan Sitki Dizdar.

**Visualization:** Hasan Bakkal

**Writing – review & editing:** Osman Baspinar, Hasan Bakkal, Oguzhan Sitki Dizdar.
